# Effects of prescription adaptation by pharmacists

**DOI:** 10.1186/1472-6963-10-313

**Published:** 2010-11-17

**Authors:** Michael R Law, Steven G Morgan, Sumit R Majumdar, Larry D Lynd, Carlo A Marra

**Affiliations:** 1 Centre for Health Services and Policy Research, School of Population and Public Health, The University of British Columbia. 201-2206 East Mall, Vancouver, BC, V6T 1Z3, Canada; 2 Centre for Health Services and Policy Research, School of Population and Public Health, The University of British Columbia. 201-2206 East Mall, Vancouver, British Columbia, V6T 1Z3, Canada; 3 Division of General Internal Medicine, Department of Medicine, University of Alberta. 2E3.07 Walter Mackenzie Centre, Edmonton, AB, T6G 2B7, Canada; 4 Collaboration for Outcomes Research and Evaluation (CORE), Faculty of Pharmaceutical Sciences, The University of British Columbia and Centre for Health Evaluation and Outcome Sciences, Providence Health Care Research Institute. 2146 East Mall, Vancouver, British Columbia, V6T 1Z3, Canada

## Abstract

**Background:**

Granting dispensing pharmacists the authority to prescribe has significant implications for pharmaceutical and health human resources policy, and quality of care. Despite the growing number of jurisdictions that have given pharmacists such privileges, there are few rigorous evaluations of these policy changes. This study will examine a January 2009 policy change in British Columbia (BC), Canada that allowed pharmacists to independently adapt and renew prescriptions. We hypothesize this policy increased drug utilization and drug costs, increased patient adherence to medication, and reduced total healthcare resource use.

**Methods/Design:**

We will study a population-based cohort of approximately 4 million BC residents from 2004 through 2010. We will use data from BC PharmaNet on all of the prescriptions obtained by this cohort during the study period, and link it to administrative billings from physicians and hospital discharges. Using interrupted time series analysis, we will study longitudinal changes in drug utilization and costs, medication adherence, and short-term health care use. Further, using hierarchical modelling, we will examine the factors at the regional, pharmacy, patient, and prescription levels that are associated with prescription adaptations and renewals.

**Discussion:**

In a recent survey of Canadian policymakers, many respondents ranked the issue of prescribing privileges as one of their most pressing policy questions. No matter the results of our study, they will be important for policymakers, as our data will make policy decisions surrounding pharmacist prescribing more evidence-based.

## Background

### Context

Access to primary care is an important concern for patients in almost every jurisdiction examined. In Canada, nearly 4 million individuals report not having a regular physician and over 2 million report difficulties in accessing routine or ongoing care [1]. For at least some of these individuals, and for certain components of primary health care, non-physician health professionals may represent high-quality alternatives. There is some research evidence on the potential of deploying pharmacists in primary care [2]; that literature, and practical experience in other jurisdictions points to a considerable amount of untapped pharmacist human resources [3-7]. Expanding the scope of pharmacy practice may be a cost-effective way to enhance patient access and adherence to medicines, and to reduce the clinical burden on primary care physicians.

Around 53% of Canadians fill one or more prescriptions each year, and at least half of these represent chronic medications used to manage cardiovascular risk factors [8,9]. However, rates of continuous use of medicines for chronic conditions are often sub-optimal [10]. But with prescription lengths limited to approximately 3 months in most provinces (with up to 4 refills if they are provided), access to primary care doctors for the purpose of prescription renewal may be a barrier to continuous adherence to long-term drug therapies. Thus, granting pharmacists prescribing authority may increase Canadians' access to medications. Though pharmacists are highly trained in matters related to the effects, interactions, and appropriate use of medicines, their expertise is seldom called upon as a first-line primary health care provider in community settings.

Recently, numerous Canadian provinces have implemented programs designed to expand the scope of pharmacy practice. The first province to move in this direction was Alberta, which implemented a program in 2007 that allows pharmacists to prescribe medications and adapt existing prescriptions [3]. One year later, three-quarters of pharmacists in the province reported that they regularly renewed or adapted prescriptions [11]. Several other provinces allow pharmacists similar prescribing privileges, and the remaining provinces have passed the enabling legislation to allow pharmacist prescribing in the future. The trend towards pharmacist prescribing is present internationally as well. The United Kingdom has introduced "independent prescribing", which gives pharmacists the ability to prescribe all medications after completing a training program. Likewise, in the United States, collaborative drug therapy management by pharmacists is permitted by the federal government and by at least 40 individual states [3]. All of these changes granting pharmacists prescribing authority may have significant implications for quality of care.

### Policy Change: A Natural Experiment

A January 2009 policy change in the Canadian province of British Columbia (BC) provides an opportunity to generate valuable information about the impacts of changes in pharmacists' prescribing authority. This policy allowed pharmacists to adapt existing prescriptions without the consent of the original prescriber [12]. The policy was adopted based on the potential benefit of increased patient adherence to medicines and an expected reduction in the visit burden on primary care physicians [13]. Prescription adaptations include changing the dose, regimen, or formulation of a prescription, renewing an existing prescription and making therapeutic substitutions to an alternative drug from the same class. Therapeutic substitutions are only permitted in a limited number of drug classes: histamine 2 receptor blockers, non-steroidal anti-inflammatory drugs, nitrates, angiotensin converting enzyme inhibitors, dihydropyridine calcium channel blockers and proton pump inhibitors. Further, pharmacists cannot modify the dose or regimen for prescriptions treating cancer, cardiovascular disease, asthma, seizures or psychiatric conditions.

Pharmacist-initiated renewal of a prescription involves their dispensing of medicines beyond the term specified in the original prescription. BC pharmacists are only permitted to make renewals under certain circumstances. Renewals can only be made for patients with long-term chronic conditions who have been on the same therapy for more than six months. Further, pharmacist-initiated renewals may only occur within the first six months after an original prescription is filled. Pharmacists cannot renew prescriptions for narcotics and psychiatric medications. Finally, pharmacists are required to notify the original prescriber of a renewal or adaptation within 24 hours, and, although under no obligation to participate, pharmacists who do so must adhere to guidelines and possess two million dollars of personal liability coverage.

Despite the fact that a growing number of jurisdictions are giving pharmacists various forms of prescriptive privileges, there are conspicuously few rigorous evaluations of these policy changes. Allowing pharmacists to modify or renew prescriptions may or may not improve treatment quality and continuity of care by improving drug selection, dosing, and use [14] - this is a testable hypothesis that has as yet not been convincingly investigated. Therefore, we undertook a study to evaluate pharmacist prescribing in BC. We hypothesized that prescription adaptation by pharmacists increased drug utilization and drug costs, increased patient adherence to medication, reduced the use of ambulatory physician services, and had no effect on hospitalization rates.

## Methods/Design

### Study Setting and Population

British Columbia (BC) is Canada's westernmost and third most populous province, with nearly 4.5 million residents in 2009 [15]. Approximately half of the population lived in the greater Vancouver area [16]. Our study will focus on a population-based open cohort of BC residents. In 2006, 14.7% of the population were over 65 and 50.3% were female [17]. The population is ethnically diverse, with significant numbers of people identifying as Europeans, Aboriginals, East Asians, and South Asians [18]. Thus, the overall population is typical of that seen in the larger provinces of Canada and is reasonably generalizable to many North American jurisdictions. For all individuals, we will extract data on their drug utilization as well as a range of control variables. Based on prior work with the BC databases, we anticipate an overall cohort of over 4 million individuals, which includes the majority of BC residents, with the exception of First Nations and veterans who receive drug benefits through the Federal Government.

Health care is provided to all residents through the BC Medical Services Plan, which covers medically necessary physician and hospital services. Unlike hospitals and physicians, prescription drug coverage is a mix of public and private programs. Public coverage is available to all residents through the Fair PharmaCare program, which covers prescription drugs after households have met an income-based deductible. The public program uses several cost containment mechanisms, including a formulary of reimbursable medications and reference-based pricing for several drug classes. Estimated provincial health expenditure in 2009 was $23.4 billion, about $2.6 billion of which paid for prescription drugs [19,20]. In sum, this public expenditure represented about 42% of total drug costs [20]. Alongside this public coverage, many BC residents hold private drug coverage, largely paid for through their employer.

To ensure maximum policy relevance, the team will maintain regular communication with local and national decision makers. This research project was developed in collaboration with the BC Ministry of Health Services and upon consultation with the BC Pharmacy Association, so we expect the results will affect future policy development or the refinement of existing policies. This study was reviewed and approved by the University of British Columbia Behavioural Research Ethics Board.

## Data Sources

### Data Access and Linkage

Three population-based, administrative data sources will be used in this analysis: (1) the BC PharmaNet prescription drug claims database, and (2) health services utilization databases held at Population Data BC. We plan to study data for a period of 5 years prior to the policy and 2 years after (January 1, 2004 through December 31, 2010).

### The Databases

#### BC PharmaNet

Pharmaceutical dispensing data come from the prescription monitoring system-BC PharmaNet-that was established in 1995 to track progress toward deductibles under the BC PharmaCare program, and to monitor for contraindicated combinations of prescribed drugs. It contains a comprehensive record of ambulatory prescriptions received by all residents of the province. The PharmaNet database will be used to capture data on individual prescriptions, including drug type, total cost, whether the prescription is a physician-initiated refill, and whether the prescription was adapted or renewed by a pharmacist. The design of the PharmaNet database makes it possible to 'nest' all prescriptions according to the originating pharmacy; this information will be used to determine the characteristics of the originating pharmacy such as the volume of prescriptions. These databases have been used extensively in pharmaceutical policy and pharmacoepidemiology research, and are considered to be among the most valid and highest quality available in Canada [21-23].

#### Health Services Data from Population Data BC

Health services data holdings of Population Data BC include administrative data files containing information from medical service claims submitted by physicians, hospital separations, and vital statistics (births/deaths). Specific information to be drawn from these data sources for the purposes of this project will include the following: from the Medical Services Plan registration files, descriptive information about individuals in the study population (age, sex, Local Health Area, duration of residency); from the Medical Services Plan payment file, medical services used and data for the construction of profiles of patient morbidity ICD-9 codes); from the Hospital Separations files, data for the construction of measures of patient morbidity and clinical complexity (discharge data and up to 25 primary and secondary ICD-10 diagnosis codes); and from the Vital statistics, date of death.

#### Income Data

For each individual, we will assign income percentiles using existing custom tabulations of Statistics Canada's Taxfiler Database at the Census Dissemination Area level [24]. In previous studies, these data have shown reasonable agreement with individual-level verified incomes [25].

## General Work Plan

**Figure 1:**
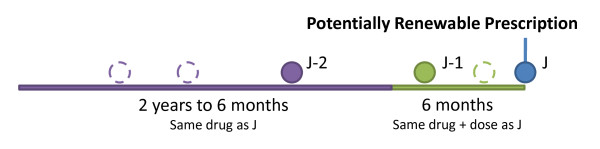
**Definition of a "Potentially Renewable Prescription" (J)**. The solid circles are examples of prescriptions that must have been filled: one prior to six months before the potentially renewable one (J-1) and one in the 18 months prior to that (J-2). The outlines represent examples of other possible prescriptions during the same time periods.

For various components of the analysis, we will use specific subsets of prescription drug claims. First, we will use data on all prescriptions to analyse the prevalence and characteristics of pharmacist adaptations and renewals and their overall impact on drug utilization and costs. Second, we will study all prescriptions from drug classes in which adaptation was permitted. Third, we will determine prescriptions that were 'potentially renewable'. The guidelines for pharmacist renewal state that a prescription may be renewed for a medication used for a chronic condition that the patient has used for more than 6 months with no changes. Thus, we will define a 'potentially renewable' prescription-one that a pharmacist could have hypothetically renewed-using the following criteria (see Figure 1):

1. At least one prescription for the same drug during the period from 2 years prior to six months prior, to establish long-term "consistent use" (prescription J-2)

2. One or more prescriptions for the *same drug *during the 6 months prior to the current prescription (prescription J-1)

3. The potentially renewable prescription, prescription J, is for the *same drug *at the *same dose *as prescription J-1.

### Subgroup Analyses

To study the impact of prescription renewal on adherence to therapy and short-term health care use, we will construct cohorts of patients established on specific medications. We will study users of medication classes that are typically used continuously, such as anti-hypertensives, and are amongst the top 20 classes of drugs in terms of the number of users in BC [26]. We will exclude medication classes that pharmacists are restricted from renewing or where pharmacist renewal is unnecessary given existing dispensing rules. Based on data from the *BC Rx Atlas, 2nd Edition*, Table 1 shows the approximate numbers of unique users of specific drug classes to be studied [26].

**Table 1:** Number of unique users in potential drug classes for analysis in 2006

*Drug Class*	*Unique users in 2006*
Cardiovascular: Antihypertensives	587,814
Psychiatric: Antidepressants	382,813
Acid reducing drugs	316,738
Cardiovascular: Statins	284,364
Hormonal contraceptives	204,629
Diabetes: Oral drugs (non-insulins)	128,408
Bisphosphonates	100,944
Cardiovascular: Antithrombotics	82,136

### Variables of Interest

#### Prescription Rate

Our analysis of drug utilization will focus on both the number of Defined Daily Doses (DDDs) of particular medications and the number of days supplied. Days supply is a field that must be entered by pharmacists in the PharmaNet database for the prescription dispensation to be adjudicated under the universal, income-based drug benefit program. DDDs are a WHO-developed measure that standardizes drug therapy and provides a consistent measure of drug use over time between therapies with different typical dosing levels [27]. Using DDDs will allow us to assess the validity of the days supplied field.

#### Costs

We will examine the cost of pharmacist adaptations and renewals by studying the drug cost per eligible patient, along with the professional fees paid to pharmacists for performing adaptations and renewals. For adaptations, we will analyze any changes in the cost per day of treatment within specific therapeutic classes. Of particular interest will be any shifts toward use of less expensive alternative drugs under BC PharmaCare's Reference Pricing Program [28-30]. For renewals, we will examine the impact on drug costs due to increased drug adherence.

#### Medication Adherence

We will analyse prescription adherence using two commonly used measures [10]. The first measure is the proportion of days covered, which is the number of days supply of medication divided by the number of days in each study month. Second, we will construct measures of persistence, defined as the number of days of therapy without a gap of 90 or more days.

#### Ambulatory physician visits

To assess the impact of the policy changes in the visit burden on primary care physicians, we will analyse the number of ambulatory physician care visits per patient per month, for patients prescribed each renewal or adaptation-eligible class of drug. While it would be ideal to study visits that were only intended to renew a prescription, billing data do not contain sufficient detail.

#### Hospital Admissions

To assess any changes in hospitalization rates, we will analyse the number of hospital admissions for relevant diagnoses (i.e. myocardial infarction for antihypertensive users).

### Other Variables

#### Demographic characteristics

For modelling the determinants of prescription adaptation and renewal, we will include indicators for patient age, sex, urban/rural, and socioeconomic status (income level). To define urban and rural areas, we will divide BC Local Health Areas (LHAs) based on population density using existing publically available data [31]. Income level will be derived from Statistics Canada data, as described above.

#### Case-mix Adjustment

We will adjust for case-mix in resource consumption at the individual level in our models using the John's Hopkins ACG Case-Mix System [32].

#### Pharmacy and Pharmacist prescribing volume

For each pharmacy and pharmacist in our sample, we will calculate average prescribing volumes over the study period by dividing the total number of prescriptions by the number of months.

## Proposed Statistical Analysis

### Descriptive Characteristics of Adaptations

We will use the complete population-based cohort of prescriptions to detail the following descriptive characteristics of adaptations for the first year of the program:

1. Number and types of adaptations performed;

2. Which drug classes and particular agents were most regularly adapted;

3. The concentration of adaptations amongst particular pharmacists, particular pharmacies and particular geographic regions;

4. The cost of professional services fees for the adaptation program.

### Impact of Pharmacist Adaptation

Using Interrupted Time Series Analysis, we will study longitudinal changes in drug utilization and costs, medication adherence, and ambulatory care visits and hospitalizations during each study month [33]. Our models will take the following form to model each measure in month i:

$$outcome_{i}=\beta_{0}+\beta_{1}\cdot time_{1}+\beta_{2}\cdot policy_{i}+\beta_{3}\cdot policy_{i}\cdot time_{i}+\varepsilon_{i}$$

Where time represents the month in study time (i.e. 1, 2, 3...) and policy is an indicator variable indicating if the policy is in effect at that observation point. The two parameters of interest are β2, which indicates any immediate change in the level of the outcome and β3, which indicates any change in the trend of the outcome following initiation of the policy. As the monthly observations may be correlated over time, we will control for autocorrelation using appropriate adjustments in a generalised least squares model, or a similar model for correlated data [33]. We will use the resulting model estimates to calculate both absolute and percentage changes in the outcomes to make the results easily interpretable [34].

As the policy may have had differential uptake rates amongst particular pharmacists, we will also explore constructing a "control" cohort for this analysis in several ways. For example, we will explore the utility of stratifying our sample into two groups: patients that visited a pharmacy that adapts prescriptions, and patients that did not. This would allow us to more specifically identify an effect by having a control series in all our time series regressions and perhaps to determine if "early adoptors" are representative of the pharmacist population in general. Alternately, we may explore using propensity score matching to pair individuals who had a prescription adapted with otherwise similar individuals who did not [35] or instrumental variables analysis.

### Determinants of Pharmacist Adaptations and Renewals

We will use multi-level modelling techniques to determine what factors at the region, pharmacy, patient and prescription levels are associated with prescription adaptations and renewals. In particular, we will investigate systematic differences in how the policy has affected patients of different age, sex and socioeconomic status. For example, we know that drug use varies by sex; for instance, there are higher rates of antidepressant use amongst women in Canada [36]. Thus, it may be that this policy has differential effects on men and women. Further, one identified goal of the policy was to increase access to medicines in rural communities. Thus, we will investigate rural region as a key factor.

By using a hierarchical model to model the probability of an adaptation or renewal, we can estimate the effect of different region, pharmacy, and patient characteristics while controlling for the clustering of observations within these different levels. For the determinants associated with adaptation, we will use the dataset containing all prescriptions for drugs potentially subject to adaptation. For renewals, we will use the dataset of potentially renewable prescriptions described above.

Our model will contain a hierarchical structure of 4 levels and include the following variables:

• Regional characteristics (of ~79 regions with sufficient population size): urban/rural; primary care physician supply (per 10,000 population); average income

• Pharmacy characteristics: chain/independent; number of pharmacists; volume of prescriptions, patients, and prescribers

• Patient characteristics: patient age, sex, health status, income

• Prescription characteristics: drug class; length of prescription; calendar month (to control for seasonality)

When modelling, we will test intra-cluster correlation coefficients to determine which levels of the hierarchical structure are important.

In both analyses, we will control for clustering at each level by appropriately including random effects terms in the model. As the primary goal of our modelling strategy is to test for differences between types of LHAs (i.e. urban/rural) and not for differences between individual LHAs, we have chosen to use random effects rather than fixed effects models. Our models will thus have the following general structure to model the probability of adaptation or renewal:

$$Pr{\left(adaptation=yes\right)}_{ijk}=\;\beta_{0}+\beta_{1}X_{i}+\;\beta_{2}X_{j}\;+\beta_{3}X_{k}\;+\;b_{i}\;+\;b_{j}+\;b_{k}+\varepsilon_{ijk}$$

where *X_i_*, *X_j_*, *X_k _*and *X_l _*are vectors of variables associated with LHA *i*, pharmacy *j*, patient *k *and prescription *l*, respectively, and *b_i_*, *b_j_*, and *b_k _*are random effects terms to account for multiple prescriptions from the same LHA *i*, pharmacy *j *and patient *k*.

## Discussion

Using a prospective population-based cohort study and interrupted time series analyses, we plan to take advantage of a natural experiment in policy change with respect to pharmacist adaptation and renewal. Short of a randomized trial, our proposed approach has perhaps the highest level of internal validity that could be brought to bear to answer such an important policy question. Our analysis will benefit from having comprehensive population-based data on every prescription for the majority of the BC population, something that is not possible in other Canadian jurisdictions with similar policies in place. Despite its novelty and strengths, we recognize several limitations.

### Study Limitations

First, while our study methodology is based on a strong quasi-experimental research design, individual patients could not feasibly be randomized to treatment (as in a controlled trial). While it might have been possible to randomize the pharmacists themselves to when they could begin using their advance prescribing privileges, this was not part of the policy implementation. Second, our study will be limited to administrative health data, and as such will not contain detailed clinical data that might be obtained through medical records. Third, we will have limited measures regarding the appropriateness of prescribing and patient or physician or pharmacist satisfaction. Finally, we will not have access to data on over-the-counter medications use. This will only be a potential issue for the few drug classes where there are over-the-counter equivalents, such as acid-suppressing drugs. However, as many individuals have low deductibles under BC's Fair PharmaCare program, the prescription alternative will be less costly for many individuals with regular drug use.

## Conclusion

In a recent survey of Canadian policymakers, many respondents from across the country ranked the issue of prescribing privileges as one of their most pressing policy questions [37]. No matter the results of our study, they will be important for policymakers. If we find the policy is safe and effective and cost-neutral, it will provide justification for widespread adoption elsewhere and provide justification for it's continuation in BC. If it leads to substantially increased drug costs or health care use, it may need to be significantly altered or abandoned. However, our study findings will make such decisions evidence-based, and help inform other healthcare jurisdictions.

## Competing Interests

Drs. Law, Morgan and Majumdar declare that they have no competing interests. Both Drs. Lynd and Marra have received research funding from the BC Pharmacists Association to investigate pharmacy adaptation services in BC.

## Authors' contributions

All authors contributed to the conception and design of the study. ML drafted the manuscript and all authors participated in revisions for intellectual content. All authors read and approved the final manuscript.

## Pre-publication history

The pre-publication history for this paper can be accessed here:

http://www.biomedcentral.com/1472-6963/10/313/prepub
